# Associations of physical activity and sedentary behavior with chronic low back pain in middle-aged and older adults: a cross-sectional study

**DOI:** 10.3389/fpubh.2026.1782597

**Published:** 2026-02-19

**Authors:** Haidong Gu, Jing Hou, Xinghuo Fu, Jiyue You

**Affiliations:** 1 Binhai County People's Hospital, Yancheng, China; 2 The People’s Hospital of Aba Tibetan and Qiang Autonomous Prefecture, Sichuan, China; 3 Taizhou No.4 People's Hospital, Taizhou, China; 4 Sichuan Academy of Medical Sciences & Sichuan Provincial People's Hospital (SAMS&SPPH, Affiliated Hospital of UESTC), Chengdu, China

**Keywords:** activity, aging, epidemiology, low-back-pain, sedentary

## Abstract

**Objective:**

To investigate the associations between physical activity levels, sedentary behavior, and the risk of chronic low back pain (CLBP) among middle-aged and older adults.

**Materials and methods:**

A cross-sectional survey was conducted among 472 community-dwelling adults aged ≥45 years recruited from communities in Binhai County, Yancheng City, Jiangsu Province, China. Physical activity and sedentary behavior were assessed using the IPAQ-short form. CLBP was defined as low back pain occurring almost daily for ≥3 months. Sociodemographic, lifestyle, and health-related factors were collected as covariates. Associations were examined using chi-square tests and multivariable logistic regression models.

**Results:**

The prevalence of CLBP was 16.1%. Individuals who met physical activity guidelines had a significantly lower risk of CLBP (adjusted OR = 0.218, 95% CI: 0.116–0.410). Sedentary behavior was independently associated with a higher risk of CLBP (adjusted OR = 2.720, 95% CI: 1.562–4.738). Stratified analyses showed consistent associations across subgroups defined by sex, residence, marital status, lifestyle factors, and chronic diseases, with no significant interaction effects.

**Conclusion:**

Insufficient physical activity and sedentary behavior are major modifiable risk factors for CLBP in middle-aged and older adults. Replacing sedentary time with regular moderate-intensity physical activity and interrupting prolonged sitting may be effective strategies for CLBP prevention and healthy aging.

## 1 Introduction

With the acceleration of global population aging, the health issues of middle-aged and older individuals have gradually become a focus of societal concern. Chronic low back pain (CLBP) is one of the most common musculoskeletal disorders among middle-aged and older people, characterized by high prevalence, high recurrence rate, and high disability rate. According to a report by the World Health Organization (WHO), approximately 70 to 80% of people worldwide experience varying degrees of back pain at some point in their lives, with the prevalence of chronic low back pain in the middle-aged and older population being even higher, reaching 30 to 40% (1).

Chronic low back pain not only affects physical function but also significantly reduces the quality of life and social participation of middle-aged and older individuals. Studies have shown that long-term back pain leads to activity limitations, decreased balance ability, and increased risk of falls in older people (2). Moreover, chronic pain is often associated with sleep disturbances, anxiety, and depression, forming a vicious cycle of “pain—immobility—functional decline,” which poses a threat to both the physical and mental health of the older (3). Chronic low back pain also results in substantial economic and social burdens. Globally, it is one of the leading causes of work absenteeism and disability, with healthcare and productivity losses in the United States alone exceeding $100 billion annually (4).

Among the numerous risk factors influencing CLBP, physical activity (PA) and sedentary behavior (SB) are two key modifiable lifestyle factors. Adequate physical activity can reduce the risk of developing low back pain or alleviating its symptoms by strengthening the core muscles, improving spinal stability, enhancing blood circulation, and reducing inflammatory responses (5). For example, Heuch et al. (6) conducted a long-term follow-up study of 4,661 adults and found that moderate-intensity physical activities—such as brisk walking, cycling, and light household chores—significantly reduced the incidence of chronic low back pain. For middle-aged and older adults, appropriate aerobic exercise and muscle-strengthening training can effectively delay muscle degeneration and maintain joint flexibility, which are essential for spinal health.

However, modern lifestyles have increasingly placed older adults at dual risk of low physical activity and high sedentary behavior. Due to mobility limitations and reduced social engagement, older adults are more likely to adopt a sedentary lifestyle. Sedentary behavior is generally defined as any waking behavior performed in a sitting or reclining posture with an energy expenditure of ≤1.5 metabolic equivalents (METs) (7). Numerous studies have confirmed that prolonged sedentary behavior can lead to atrophy of the back muscles, impaired intervertebral disc nutrition, and decreased metabolic function, thereby increasing the risk of low back pain (8). More importantly, sedentary behavior also indirectly contributes to the persistence and recurrence of chronic pain by promoting obesity, insulin resistance, and systemic inflammation.

Although previous studies have separately investigated the effects of physical activity and sedentary behavior on CLBP, their findings remain inconsistent. Some studies have shown that moderate-intensity physical activity exerts a protective effect, whereas excessive or high-impact exercise may increase spinal loading and aggravate pain (9). Similarly, the relationship between sedentary behavior and low back pain remains controversial. While some researchers have reported that longer sedentary time is associated with a higher risk of CLBP (10), others have suggested that the harmful effects of sedentary behavior are modulated by multiple factors such as posture, rest frequency, and muscle endurance (11). However, most existing studies have been descriptive in nature, and the interaction between physical activity and sedentary behavior in the context of CLBP, especially in middle-aged and older populations, remains underexplored. Furthermore, previous research has not sufficiently addressed the individual variability in exercise sensitivity and adherence among older adults, particularly in the presence of degenerative changes and comorbid chronic diseases. This gap in the literature underscores the novelty of our current study, which aims to examine how physical activity and sedentary behavior jointly influence CLBP risk in this population, with an emphasis on tailored health promotion strategies.

Therefore, the objective of this study was to examine the associations between physical activity levels, sedentary behavior, and the risk of CLBP among community-dwelling middle-aged and older adults from Binhai County, China. We hypothesized that insufficient physical activity would be associated with a higher risk of CLBP, whereas meeting recommended physical activity guidelines would be associated with a lower risk. In addition, we hypothesized that sedentary behavior would be independently associated with an increased risk of CLBP, even after adjustment for potential confounding factors.

## 2 Methods

### 2.1 Study design

This study adopts a cross-sectional survey design, aimed at exploring the associations between physical activity levels, sedentary behavior, and the risk of CLBP in middle-aged and older individuals. The entire research process strictly follows the STROBE (STrengthening the Reporting of OBservational studies in Epidemiology) guidelines to ensure the scientific rigor and standardization of the study methods and reporting (12). The study protocol was reviewed and approved by the Ethics Committee of Binhai County People’s Hospital (Ethical approval number: 2025BYLL037) and conducted in accordance with the ethical principles of the Declaration of Helsinki. Prior to participation, all participants received written information, which included the study objectives, survey content, potential risks, and privacy protection measures, and provided voluntary written informed consent.

### 2.2 Study participants and inclusion/exclusion criteria

This study adopted a community-based cross-sectional design. The sample size was determined based on feasibility considerations and participant availability during the study period, rather than *a priori* hypothesis-driven sample size calculation. A total of 516 eligible community residents aged ≥45 years were initially approached, and 472 participants were included in the final analysis after applying predefined inclusion and exclusion criteria. Given the observed prevalence of chronic low back pain (24.8%) and the effect sizes identified for physical activity and sedentary behavior, the final sample size provided adequate statistical power for the primary analyses (13).

*Inclusion criteria*: (1) aged ≥ 45 years; (2) permanent residents; (3) able to understand and complete the questionnaires; (4) able to perform daily activities (excluding those who are bedridden or rely on wheelchairs for extended periods).

*Exclusion criteria*: (1) diagnosed with severe mental illness or severe cognitive impairment, unable to cooperate with the questionnaire survey; (2) history of severe trauma or in the postoperative recovery period following spinal surgery within the past 3 months; (3) presence of serious diseases that significantly impair activity (such as advanced malignancies, severe heart failure, or joint diseases with restricted movement); (4) currently undergoing systemic treatments that have a strong impact on pain (such as long-term corticosteroids, potent analgesics, or recent spinal surgery).

### 2.3 Assessment of chronic low back pain

Participants were classified as having CLBP if they reported pain in the region between the lower ribs and the buttocks, which had persisted almost daily for at least 3 months (14). CLBP was determined using the following two questions, both of which must be answered “Yes”: (1) “Have you experienced lower back pain, soreness, or stiffness almost every day for 3 months or longer?” (2) “Do you currently experience lower back pain, soreness, or stiffness?” The primary outcome variable for CLBP was assessed as a binary outcome: “presence/absence of CLBP.”

### 2.4 Assessment of physical activity and sedentary behavior

This study used a questionnaire method for both assessments: (1) Physical activity assessment: The IPAQ-short form (International Physical Activity Questionnaire Short Version) was used to collect data on walking, moderate-intensity, and vigorous-intensity activities over the past 7 days, including their frequency and duration (15). Previous validation studies have shown that the IPAQ-short form has acceptable internal consistency, with Cronbach’s alpha values exceeding 0.70 (16). These were converted into MET-minutes per week according to standard procedures. Sedentary time was recorded based on the daily sitting time item from the IPAQ. According to WHO and IPAQ recommendations, physical activity levels were categorized as “sufficient” (≥600 MET-min/week) or “insufficient” (<600 MET-min/week), and stratified analysis was conducted. (2) Sedentary behavior definition: Sedentary behavior was defined according to the Sedentary Behavior Research Network (SBRN) as sitting or lying with energy expenditure ≤1.5 METs. Data was collected on the frequency of extended periods of sitting (≥2 h continuously) during the daytime and interruptions to these sedentary periods (17). Sedentary time was grouped for comparative analysis either by quantiles or clinical thresholds.

### 2.5 Covariates and potential confounders

Covariates were collected through a structured questionnaire and included in the multivariable analysis: demographic factors (age, sex, education level, marital status, residential area), lifestyle factors (smoking, alcohol consumption), and health status (BMI, hypertension, diabetes, sleep quality). These variables were selected based on potential confounding pathways identified in previous literature related to back pain or activity levels (18–20).

### 2.6 Data collection and quality control

All investigators underwent standardized training and completed a pilot test to ensure understanding of the questionnaire and the measurement process. Paper-based or electronic questionnaires were completed by the investigators on-site or face-to-face, with intentional omissions and logical inconsistencies corrected on the spot. Data was entered twice, and consistency checks were performed, with range checks and missing value checks to ensure data quality.

### 2.7 Statistical analysis

The preliminary data collection was conducted using paper-based questionnaires, and all records were independently entered into an electronic database by trained research team members after completion. Data quality control procedures were performed prior to analysis, including logical checks, range checks, and verification of internal consistency. Records with missing or incomplete information on key variables, including physical activity, sedentary behavior, and CLBP, were excluded from the final analysis.

Data entry and management were carried out using Microsoft Excel, while statistical analyses were conducted using SPSS version 27.0 (IBM Corp., Armonk, NY, USA) and GraphPad Prism version 8.0.2. Continuous variables were assessed for normality using the Shapiro–Wilk test. Normally distributed variables were summarized as means ± standard deviations (Mean ± SD), whereas non-normally distributed variables were presented as medians with interquartile ranges (IQR). Categorical variables were described using frequencies and percentages. Comparisons of categorical variables between groups were performed using the chi-square test. For continuous variables, between-group differences were analyzed using independent-samples t tests or Mann–Whitney U tests, as appropriate, based on data distribution. Multivariable logistic regression models were constructed to examine the associations between physical activity levels, sedentary behavior, and CLBP. Chronic low back pain was treated as the dependent variable, while physical activity levels and sedentary behavior were included as the primary independent variables. Potential confounders were selected *a priori* based on previous literature and biological plausibility and included age, sex, BMI, smoking status, alcohol consumption, residential area, marital status, educational level, hypertension, and diabetes. Results were reported as adjusted odds ratios (ORs) with 95% confidence intervals (CIs).

The use of multiple software packages allowed complementary analytical functions, with SPSS primarily used for descriptive statistics and regression analyses and GraphPad Prism used for data visualization and verification of analytical results. All statistical tests were two-sided, and a *p* < 0.05 was considered statistically significant.

## 3 Results

### 3.1 Baseline characteristics of participants

**Figure 1:**
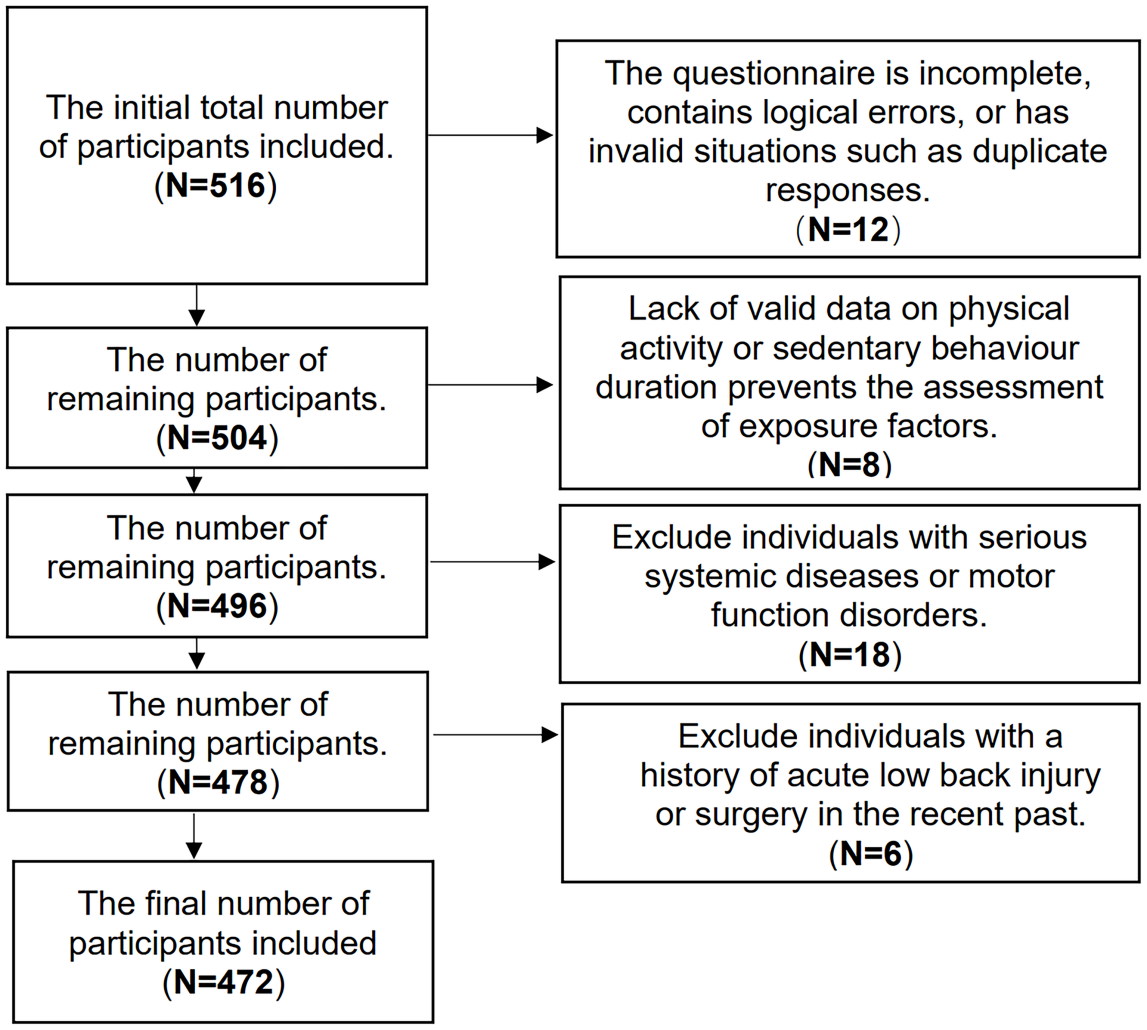
Flowchart of the sample inclusion process for the study.

A total of 516 community-dwelling middle-aged and older residents were initially enrolled in the study. After rigorous data quality control and application of the exclusion criteria, 472 participants were included in the final analysis (Figure 1). Table 1 presents the demographic and health-related baseline characteristics of the study participants. Among the 472 valid participants, the overall prevalence of CLBP was 16.1%. Specifically, 245 males (51.9%) and 227 females (48.1%) participated, with a nearly balanced male-to-female ratio. The average age of participants was 58.04 ± 10.25 years, and there was no statistically significant difference in age between the CLBP and non-CLBP groups (*p* = 0.580).

**Table 1:** Sociodemographic characteristics of sample.

Characteristic	All (*n =* 472) (weighted column %)	No *N* (%)	Yes *N* (%)	*P*
Sex				0.716
Male	245 (51.9)	207 (84.5)	38 (15.5)	
Female	227 (48.1)	189 (83.3)	38 (16.7)	
Age	58.04 ± 10.25	58.16 ± 10.42	57.45 ± 9.32	0.580
Region of residence				0.530
Countryside	111 (23.5)	91 (82.0)	20 (18.0)	
Cities	361 (76.5)	305 (84.5)	56 (15.5)	
Marital status				0.836
Married	400 (84.7)	335 (83.8)	65 (16.2)	
Other	72 (15.3)	61 (84.7)	11 (15.3)	
Education				0.074
Below secondary school	396 (83.9)	327 (82.6)	69 (17.4)	
Secondary school and above	76 (16.1)	69 (90.8)	7 (9.2)	
Smoking				0.948
No	309 (65.5)	259 (83.8)	50 (16.2)	
Yes	163 (34.5)	137 (84.0)	26 (16.0)	
Drinking				0.208
No	321 (68.0)	274 (85.4)	47 (14.6)	
Yes	151 (32.0)	122 (80.8)	29 (19.2)	
High blood pressure				0.901
No	264 (55.9)	221 (83.7)	43 (16.3)	
Yes	208 (44.1)	175 (84.1)	33 (15.9)	
Diabetes				0.802
No	420 (89.0)	353 (84.0)	67 (16.0)	
Yes	52 (11.0)	43 (82.7)	9 (17.3)	
BMI level				0.286
Light or normal	358 (75.8)	304 (84.9)	54 (15.1)	
Overweight and obese	114 (24.2)	92 (80.7)	22 (19.3)	
Sleep duration	6.51 ± 2.17	6.50 ± 2.19	6.55 ± 2.03	0.843

Regarding residential area distribution, 76.5% of participants lived in urban areas, while 23.5% lived in rural areas. The difference in CLBP prevalence between these groups was not statistically significant (*p* = 0.530). In terms of marital status, 84.7% of participants were married, while 15.3% had other marital statuses, with no significant association between marital status and CLBP prevalence (*p* = 0.836).

Regarding educational level, 83.9% of participants had education up to junior high school or lower, while 16.1% had completed high school or higher education. Notably, the prevalence of CLBP in the low education group (17.4%) was higher than that in the high education group (9.2%), but this difference did not reach statistical significance (*p* = 0.074).

Lifestyle factors indicated that 34.5% of participants had a history of smoking, and 32.0% reported alcohol consumption. Smoking status was not significantly associated with CLBP prevalence (*p* = 0.948), whereas the prevalence of CLBP was slightly higher in alcohol consumers (19.2%) compared to non-drinkers (14.6%), but this difference was not statistically significant (*p* = 0.208).

In terms of comorbid chronic diseases, 44.1% of participants had hypertension, and 11.0% had diabetes. Neither hypertension nor diabetes was significantly associated with CLBP risk (*p* = 0.901 and *p* = 0.802, respectively). Regarding BMI, 24.2% of participants were overweight or obese, and this group had a higher CLBP prevalence (19.3%) compared to those with normal or lower weight (15.1%), but the difference was not statistically significant (*p* = 0.286). In terms of sleep duration, the average sleep duration among all participants was 6.51 ± 2.17 h, with no significant difference in sleep duration between the CLBP and non-CLBP groups (*p* = 0.843).

The baseline characteristics of the study sample, including sex, age, residential area, marital status, education level, lifestyle, and chronic diseases, were well-distributed. The CLBP and non-CLBP groups were comparable on the main demographic and health indicators, providing a solid foundation for further analysis of the associations between physical activity, sedentary behavior, and CLBP.

### 3.2 Association between physical activity, sleep, and CLBP

Table 2 presents the univariate analysis results for physical activity levels, sedentary behavior, and the prevalence of CLBP in this study. Among the 472 valid participants, 55.3% did not meet the physical activity standards according to the IPAQ, while 44.7% met the recommended physical activity levels. Univariate analysis showed a significant association between physical activity levels and the risk of chronic low back pain (*p* < 0.001). Specifically, the prevalence of chronic low back pain in the insufficient physical activity group was 23.8%, significantly higher than the 6.6% in the sufficient physical activity group, suggesting that adequate physical activity may have a protective effect against chronic low back pain.

**Table 2:** A univariate logistic regression analysis of physical activity levels and sedentary on chronic low back pain.

Characteristic	All (*n =* 472) (weighted column %)	Non-chronic low back pain *N* (%)	Chronic low back pain *N* (%)	*P*
IPAQ				<0.001
Substandard	261 (55.3)	199 (76.2)	62 (23.8)	
Eligible	211 (44.7)	197 (93.4)	14 (6.6)	
Sedentary				<0.001
No	219 (46.4)	198 (90.4)	21 (9.6)	
Yes	253 (53.6)	198 (78.3)	55 (21.7)	

Regarding sedentary behavior, 53.6% of participants exhibited sedentary behavior, while 46.4% did not. The univariate analysis results indicated a significant association between sedentary behavior and the risk of chronic low back pain (*p* < 0.001). The prevalence of chronic low back pain was 21.7% in the sedentary group, significantly higher than the 9.6% in the non-sedentary group, suggesting that sedentary behavior may be an important risk factor for the development of chronic low back pain.

**Figure 2:**
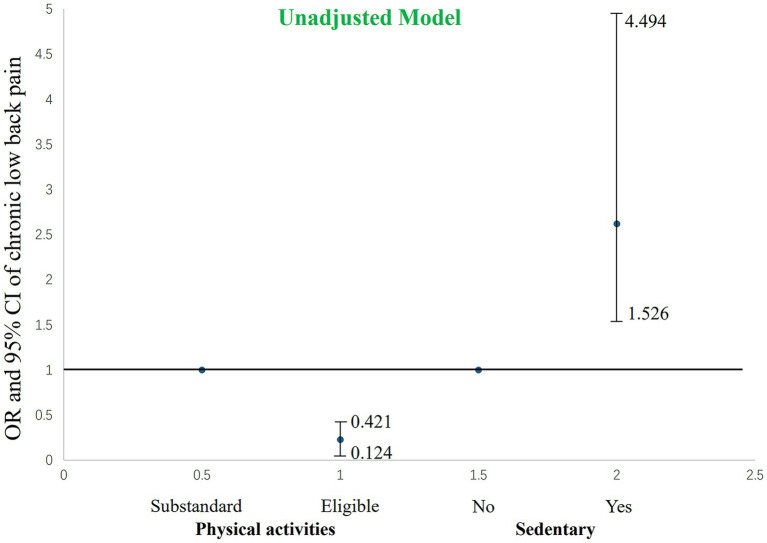
The impact of the unadjusted model’s level of physical activity and sedentary behavior on chronic low back pain.

**Figure 3:**
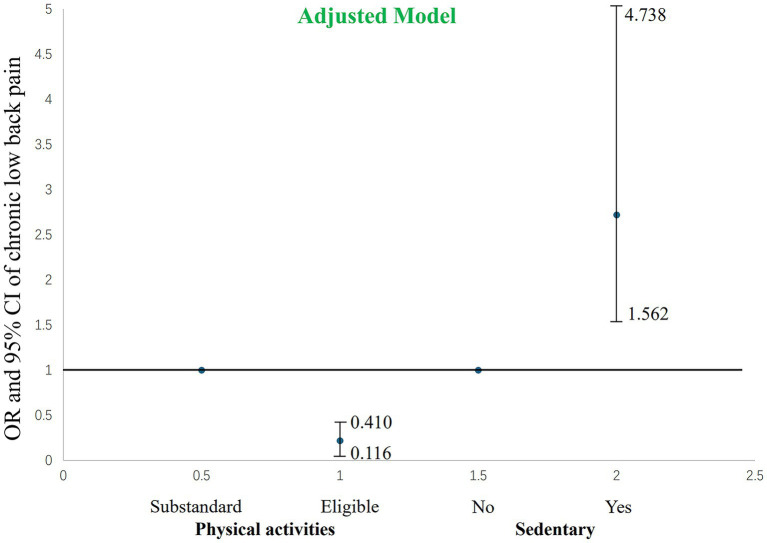
The impact of the adjusted model’s level of physical activity and sedentary behavior on chronic low back pain.

To further clarify the independent associations between physical activity levels, sedentary behavior, and chronic low back pain, a multivariable logistic regression model was constructed (Table 3 and Figures 2, 3). In the unadjusted model (Figure 2), individuals meeting physical activity guidelines had a significantly lower risk of chronic low back pain, with an OR of 0.228 (95% CI: 0.124–0.421, *p* < 0.001). This indicates that the risk of chronic low back pain in those meeting physical activity guidelines was only 22.8% of those not meeting the guidelines. In contrast, sedentary behavior significantly increased the risk of chronic low back pain, with an unadjusted OR of 2.619 (95% CI: 1.526–4.494, *p* < 0.001), indicating that individuals with sedentary behavior had a 2.6 times higher risk of chronic low back pain compared to non-sedentary individuals.

**Table 3:** Multifactorial logistic regression analysis of physical activity levels and sedentary on chronic low back pain.

Characteristic	Unadjusted model OR (95% CI)	*P*	Adjusted model OR (95% CI)	*P*
IPAQ		<0.001		<0.001
Substandard	1 (Ref)		1 (Ref)	
Eligible	0.228 (0.124–0.421)		0.218 (0.116–0.410)	
Sedentary		<0.001		<0.001
No	1 (Ref)		1 (Ref)	
Yes	2.619 (1.526–4.494)		2.720 (1.562–4.738)	

After adjusting for potential confounders including age, sex, BMI, smoking, alcohol consumption, hypertension, and diabetes (Table 3; Figure 3), the associations between physical activity levels, sedentary behavior, and chronic low back pain remained statistically significant (both *p* < 0.001). In the adjusted model, individuals meeting physical activity guidelines had an even lower risk of chronic low back pain, with an OR of 0.218 (95% CI: 0.116–0.410), further enhancing the protective effect. The adjusted OR for sedentary behavior was 2.720 (95% CI: 1.562–4.738, *p* < 0.001). Both the point estimate and the confidence interval were located to the right of the null value, indicating that after controlling for other confounding factors, the risk of chronic low back pain in individuals with sedentary behavior was still more than 2.7 times higher than that in non-sedentary individuals, further confirming that sedentary behavior is an independent risk factor for chronic low back pain.

The results of this study demonstrate that adequate physical activity levels provide significant protection against chronic low back pain in middle-aged and older individuals, while sedentary behavior significantly increases the risk of chronic low back pain. This association remains robust after controlling various potential confounding factors, suggesting that improving physical activity patterns and reducing sedentary time may be important intervention strategies for the prevention and management of chronic low back pain in middle-aged and older populations.

### 3.3 Stratified analysis

**Figure 4:**
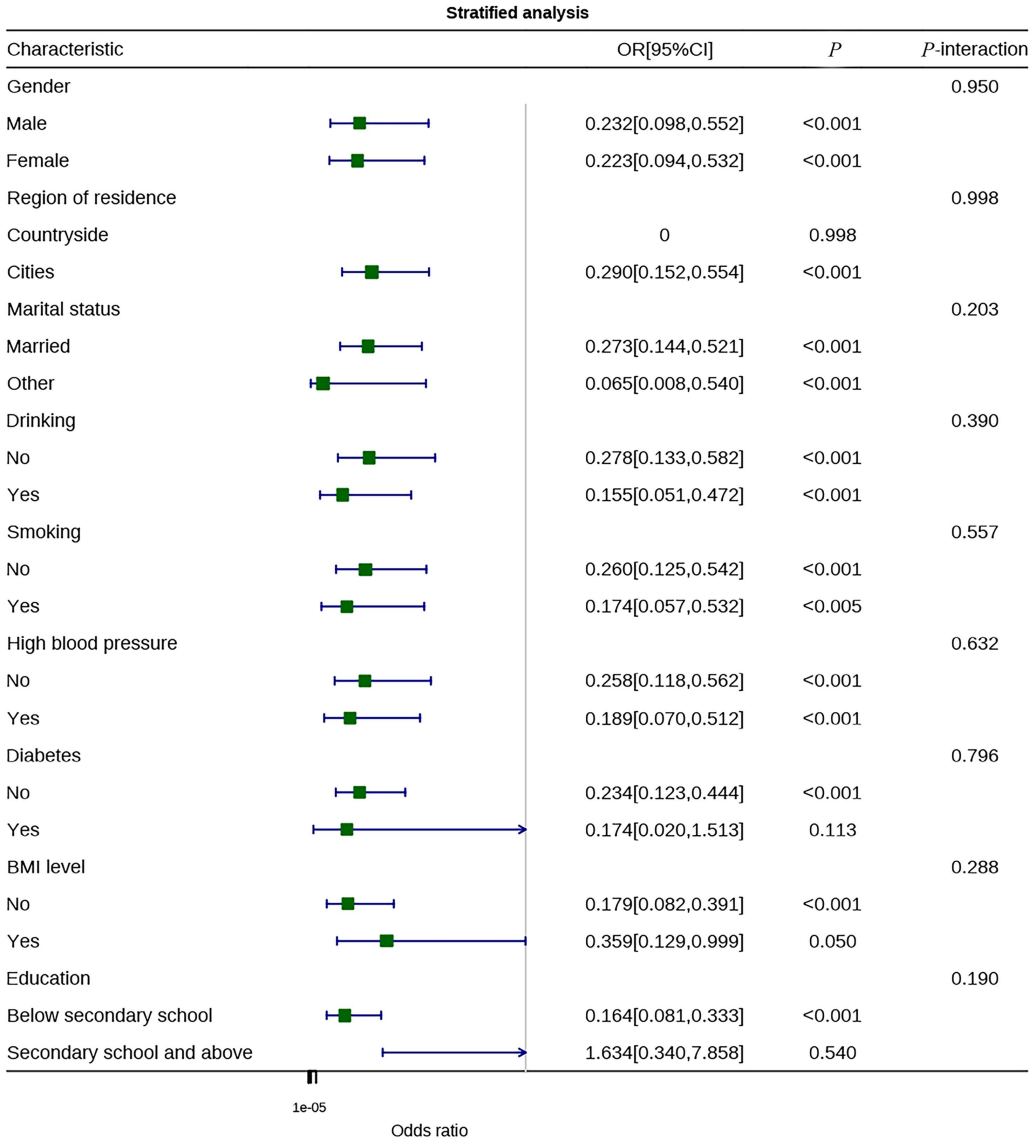
Stratified analysis of physical activity levels.

**Figure 5:**
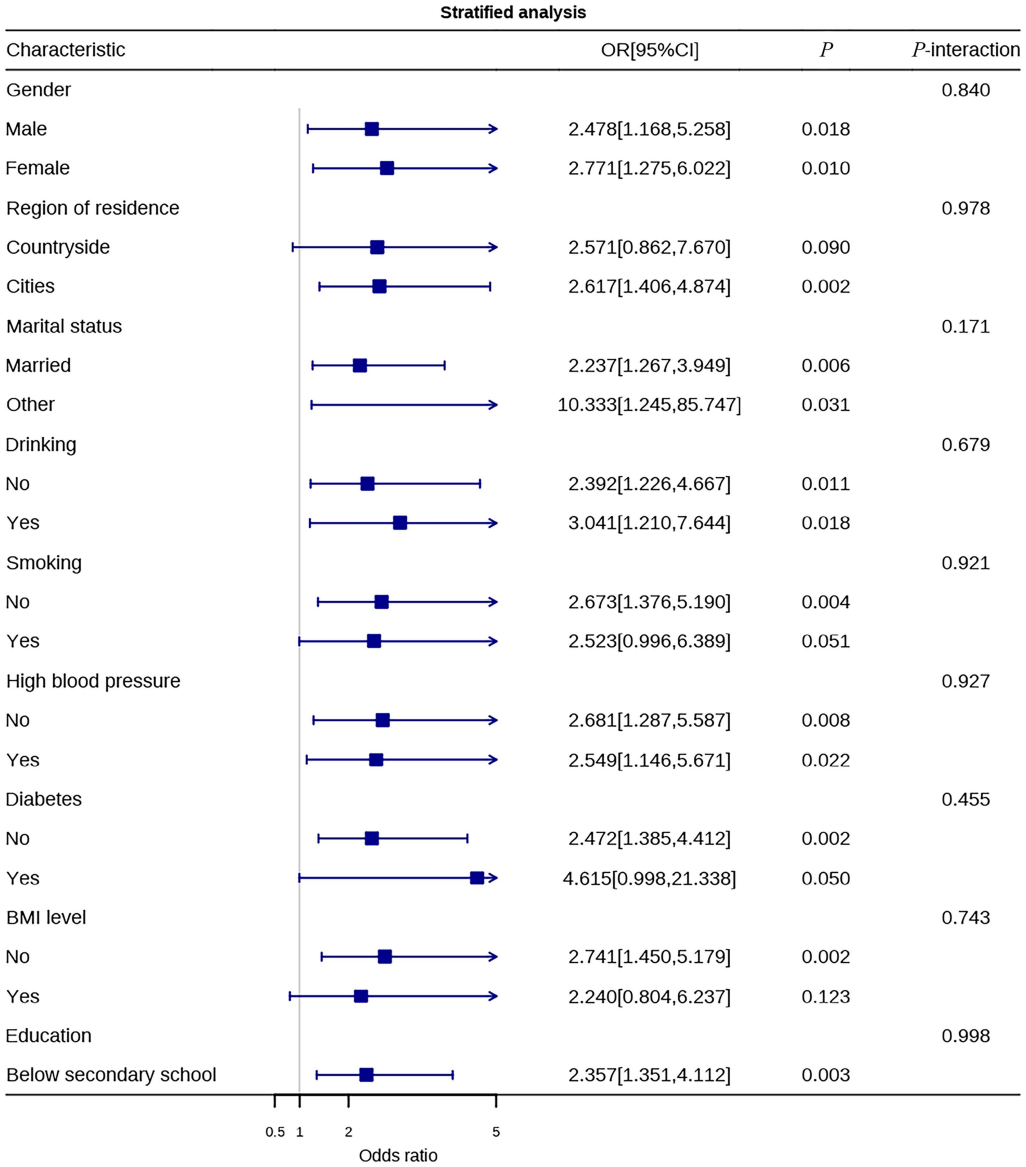
Stratified analysis of sedentary behavior.

To explore the heterogeneity in the effects of physical activity levels and sedentary behavior on CLBP, stratified analyses were conducted based on different demographic characteristics, lifestyle factors, and health status. Figure 4 shows the protective effect of physical activity across different subgroups, and Figure 5 illustrates the association between sedentary behavior and CLBP risk in various subgroups.

The stratified analysis results (Figure 4) demonstrate that meeting physical activity guidelines was significantly associated with a reduced risk of CLBP in most subgroups, with consistent protective effects across the board. In the sex-stratified analysis, both males (OR = 0.232, 95% CI: 0.098–0.552) and females (OR = 0.223, 95% CI: 0.094–0.532) showed similar reductions in risk, and the interaction effect was not significant (*P*-interaction = 0.950), indicating that the protective effect of physical activity is not influenced by sex. In the residential area stratification, urban residents who met physical activity guidelines also showed significant protection (OR = 0.290, 95% CI: 0.152–0.554), with no significant interaction effect (*P*-interaction = 0.998). Marital status stratification showed risk reductions in both married individuals (OR = 0.273) and those with other marital statuses (OR = 0.065), although the confidence interval for the latter was wide, indicating limited sample size.

Regarding lifestyle factors, meeting physical activity guidelines conferred protection both for non-drinkers (OR = 0.278) and drinkers (OR = 0.155) (*P*-interaction = 0.390); similar trends were observed for non-smokers (OR = 0.260) and smokers (OR = 0.174) (*P*-interaction = 0.557), suggesting that alcohol consumption and smoking did not significantly alter the direction of the effect of physical activity. The stratified results for chronic disease comorbidities further supported the consistent protective effect of physical activity. Both individuals without hypertension (OR = 0.258) and those with hypertension (OR = 0.189) showed significant reductions in risk (*P*-interaction = 0.632). In the diabetes stratification, the protective effect was significant in individuals without diabetes (OR = 0.234), while the trend was similar in individuals with diabetes, though not statistically significant, likely due to limited sample size (*P*-interaction = 0.796). The BMI stratification showed protection in both normal-weight individuals (OR = 0.179) and overweight/obese individuals (OR = 0.359) (*P*-interaction = 0.288). The stratification by education level showed a significant protective effect in individuals with lower education (OR = 0.164), while no significant result was found for individuals with higher education (*P*-interaction = 0.190), likely due to limited sample size in this subgroup.

The stratified analysis results (Figure 5) revealed that sedentary behavior was significantly associated with an increased risk of chronic low back pain in most subgroups, although the effect size varied between groups. In the sex-stratified analysis, sedentary behavior increased the risk for both males (OR = 2.478, 95% CI: 1.168–5.258) and females (OR = 2.771, 95% CI: 1.275–6.022), with no significant interaction effect (*P*-interaction = 0.840), suggesting that the harmful effect of sedentary behavior is not influenced by sex. In the residential area stratification, sedentary behavior significantly increased the risk for urban residents (OR = 2.617), while a similar trend was observed for rural residents, though it did not reach statistical significance (OR = 2.571, *p* = 0.090), which may be attributed to smaller sample size or differences in sedentary patterns. In marital status analysis, sedentary behavior increased risk for married individuals (OR = 2.237), while individuals with other marital statuses had an even higher OR (10.333), though with a wide confidence interval, indicating fewer stable estimates; the interaction effect was also non-significant (*P*-interaction = 0.171), suggesting cautious interpretation.

In lifestyle factors stratification, sedentary behavior significantly increased the risk of chronic low back pain in both non-drinkers (OR = 2.392) and drinkers (OR = 3.041). A similar trend was observed for smoking status, with non-smokers showing an OR of 2.673 and smokers showing an OR of 2.523, with the latter approaching statistical significance (*p* = 0.051). The interaction effects for both lifestyle subgroups were non-significant, indicating that alcohol consumption and smoking do not significantly modify the association between sedentary behavior and chronic low back pain.

In terms of comorbid chronic diseases, sedentary behavior significantly increased risk in both hypertensive (OR = 2.681) and non-hypertensive individuals (OR = 2.549). In diabetes stratification, sedentary behavior significantly increased the risk in individuals without diabetes (OR = 2.472), while the risk was even higher in those with diabetes (OR = 4.615), though with a wide confidence interval and approaching statistical significance, likely due to limited sample size. The interaction effects were not significant. The BMI stratification showed that sedentary behavior significantly increased risk in normal-weight individuals (OR = 2.741), whereas a similar trend was observed in overweight/obese individuals, but it did not reach statistical significance (OR = 2.240, *p* = 0.123). In the education level stratification, sedentary behavior significantly increased risk in individuals with education levels up to junior high school (OR = 2.357), with no significant interaction effect (*P*-interaction = 0.998).

The stratified analysis results indicate that the protective effect of meeting physical activity guidelines and the harmful effect of sedentary behavior on chronic low back pain are consistent across different subgroups based on sex, residential area, marital status, lifestyle, and health status. No significant interactions were found for any of the subgroups (all *P*-interaction values > 0.05). This suggests that the association between physical activity, sedentary behavior, and chronic low back pain is generally consistent and robust, unaffected by significant demographic characteristics or health status.

## 4 Discussion

### 4.1 Principal findings

Chronic low back pain is a prevalent musculoskeletal condition among middle-aged and older adults and represents a substantial public health challenge. In the present study, we observed a strong inverse association between meeting physical activity guidelines and the risk of chronic low back pain, as well as a positive association between sedentary behavior and chronic low back pain. These associations remained robust after adjustment for multiple demographic, lifestyle, and health-related factors.

### 4.2 Comparison with previous studies

The findings of this study regarding the relationship between physical activity and chronic low back pain align with several prospective cohort studies. A systematic review and meta-analysis by Quentin et al., found that moderate-intensity physical activity can reduce the risk of chronic low back pain by approximately 20–30%, which is consistent with the direction of risk reduction observed in this study, though the protective effect observed here was more pronounced (21). Similarly, Suh et al. found a significant association between moderate-intensity physical activity at work and reduced chronic low back pain risk, further supporting the protective role of moderate exercise (22). This study validates this effect in a middle-aged and older Chinese population, providing new evidence for the benefits of physical activity for spinal health across different cultural and regional contexts. In terms of sedentary behavior, the results of this study are highly consistent with the rapidly growing body of evidence in recent years. A systematic review by Grooten et al. highlighted a significant positive association between sedentary behavior and back pain, with sedentary individuals showing a markedly increased risk of chronic low back pain (23). A meta-analysis by Wewege et al. further quantified this relationship, showing that for every additional hour of sedentary behavior, the risk of chronic low back pain increased by about 15–20% (24). The higher risk observed in this study may reflect the increased susceptibility of the middle-aged and older population to the harmful effects of sedentary behavior, closely related to age-associated physiological changes such as muscle degeneration, reduced bone density, and metabolic decline.

The protective effect of physical activity on chronic low back pain involves multiple synergistic biological mechanisms. First, regular physical activity effectively strengthens core muscle groups, including the rectus abdominis, transverse abdominis, multifidus, and erector spinae. These muscles work together to maintain the neutral alignment of the spine, reducing the abnormal stress and shear forces experienced by the lumbar vertebrae during daily activities, thereby lowering the risk of structural damage such as intervertebral disc herniation and facet joint dysfunction (25). Second, physical activity improves local blood circulation, promoting the nutritional supply and metabolic waste clearance of lumbar and back tissues, which helps alleviate muscle tension and inflammatory responses (26). Studies have shown that exercise can reduce levels of pro-inflammatory cytokines such as tumor necrosis factor-*α* (TNF-α) and interleukin-6 (IL-6), which play crucial roles in the development of chronic pain (27, 28). Additionally, physical activity stimulates the release of endogenous opioid peptides and endorphins, which have natural analgesic effects, raise pain thresholds, and improve pain perception (29). For middle-aged and older individuals, regular exercise can also delay the progression of age-related sarcopenia, maintain joint flexibility, and preserve proprioceptive function, all of which are important physiological foundations for preventing back pain.

Sedentary behavior contributes to chronic low back pain through multiple pathophysiological pathways. Prolonged sitting places the back muscles—particularly the erector spinae and multifidus, which are essential for postural maintenance—under sustained static contraction, leading to muscle fatigue, impaired microcirculation, and the accumulation of metabolic byproducts (30). Over time, these changes result in muscle atrophy and reduced endurance. Meanwhile, the mechanical load on the lumbar spine increases substantially during prolonged sitting; under poor sitting posture, intradiscal pressure can reach 1.5–2 times that of standing. Chronic exposure to elevated disc pressure accelerates intervertebral disc degeneration, resulting in nucleus pulposus dehydration and annulus fibrosus tears, thereby increasing the risk of back pain. More importantly, sedentary behavior is closely associated with metabolic dysregulation (31). Prolonged inactivity reduces lipoprotein lipase activity, impairs triglyceride clearance, lowers high-density lipoprotein cholesterol levels, and promotes the development of obesity and insulin resistance. Obesity not only increases mechanical loading on the lumbar spine but also contributes to systemic inflammation through adipokines such as leptin and adiponectin, indirectly exacerbating chronic pain (32). In addition, sedentary behavior is often accompanied by psychological stress and sleep disturbances, forming a “pain–anxiety–inactivity–functional decline” cycle that is particularly common among older adults and substantially reduces pain management efficacy and quality of life.

### 4.3 Subgroup analyses and public health implications

The stratified analysis results provide additional insights into the associations between physical activity, sedentary behavior, and chronic low back pain across different population subgroups. Overall, the protective effect of meeting physical activity guidelines and the harmful effect of sedentary behavior were directionally consistent across subgroups defined by sex, residential area, marital status, alcohol consumption, smoking status, hypertension, and diabetes, and none of the interaction tests reached statistical significance. These findings suggest that the associations observed in the overall population are generally robust and not substantially modified by major demographic or health-related characteristics. From a public health perspective, this consistency indicates that the strategy of increasing physical activity and reducing sedentary time may be broadly applicable to middle-aged and older adults with diverse characteristics, without necessarily requiring highly complex or subgroup-specific intervention designs. Nevertheless, these implications should be interpreted cautiously, particularly when considering subgroup-specific estimates.

Importantly, several subgroup-specific results did not reach statistical significance and warrant cautious interpretation due to limited sample sizes and reduced statistical power. For example, although the protective effect of physical activity showed a consistent direction across education levels, the association was not statistically significant among individuals with higher education, who accounted for only 16.1% of the study population. Similarly, the harmful effect of sedentary behavior appeared numerically stronger among individuals with diabetes (OR = 4.615), but the wide confidence interval and borderline statistical significance likely reflect the small proportion of participants with diabetes (11.0%). Further stratification by physical activity or sedentary behavior resulted in even smaller cell sizes, which may have compromised estimation precision and increased the risk of overinterpretation. In addition, although point estimates differed across subgroups such as marital status and residential area, the absence of significant interaction effects suggests that socioeconomic and environmental factors may influence absolute activity levels rather than fundamentally altering the direction or strength of the associations. Future studies with larger and more diverse samples, particularly for underrepresented subgroups—are needed to validate these findings. Moreover, incorporating standardized measures of pain severity and functional disability, such as the Oswestry Disability Index or the Roland–Morris Disability Questionnaire, would allow a more comprehensive evaluation of the clinical relevance of physical activity and sedentary behavior in relation to chronic low back pain.

From a public health perspective, the findings of this study have important implications. On the one hand, they help clarify the role of lifestyle factors—particularly physical activity and sedentary behavior—in the development and persistence of CLBP and provide epidemiological evidence for identifying high-risk groups among middle-aged and older adults. On the other hand, these results offer a scientific basis for health management strategies, such as developing graded exercise prescriptions, optimizing daily activity patterns, and increasing standing or light-intensity activities, to facilitate early prevention and effective management of CLBP. By promoting adequate physical activity and reducing sedentary time, it may be possible not only to alleviate pain and enhance functional independence, but also to extend healthy life expectancy and reduce the broader societal and economic burden associated with chronic low back pain.

### 4.4 Limitations

Nevertheless, several limitations of this study should be acknowledged. The primary limitation is the cross-sectional design, which prevents establishing temporal or causal relationships between physical activity, sedentary behavior, and chronic low back pain. Although existing biological evidence and prospective cohort studies support the protective effects of physical activity and the harmful effects of sedentary behavior, reverse causation remains possible—that is, individuals with pre-existing chronic low back pain may reduce physical activity and increase sedentary time due to pain. Second, this study relied on self-reported questionnaires for assessing physical activity and sedentary behavior, which may introduce recall bias and social desirability bias, potentially leading to overestimation of activity levels and underestimation of sedentary time. Although the IPAQ has been widely validated, objective measurement tools would provide more accurate data. Third, this study did not include detailed assessments of specific activity types, intensity gradients, or sedentary posture, all of which may influence the risk of CLBP. Fourth, while participants were recruited from multiple communities within Binhai County, including both urban and rural residents, which enhanced the representativeness of the sample with respect to the local middle-aged and older population, the sample was still drawn from a single region and was relatively small. Therefore, caution is warranted when generalizing the findings to other populations or cultural contexts. Finally, this study did not include quantitative measures of pain severity or functional disability. Future studies could incorporate tools such as the Roland–Morris Disability Questionnaire or the Oswestry Disability Index to provide a more comprehensive evaluation of how lifestyle factors influence the severity of chronic low back pain.

## 5 Conclusion

This study confirms that insufficient physical activity and sedentary behavior are significant modifiable risk factors for chronic low back pain (CLBP) in middle-aged and older individuals. Adequate physical activity provides significant protection against CLBP, while sedentary behavior independently increases the risk of developing the condition. The effects of both factors were highly consistent across various population subgroups. These findings provide important epidemiological evidence for developing prevention strategies for CLBP in middle-aged and older populations. Improving lifestyle patterns, particularly promoting a “reduce sitting, increase movement” approach, should be a central component of primary prevention efforts for chronic low back pain.

It is recommended that physical activity promotion and sedentary behavior management be integrated into older health services. Through multi-level interventions such as health education, environmental support, and professional guidance, middle-aged and older individuals can be helped to establish and maintain healthy activity patterns. This will not only reduce the prevalence of chronic low back pain, improve functional status and quality of life, but also alleviate the related social and healthcare burden, contributing to the achievement of healthy aging goals. Given the limitations of cross-sectional study design, future research through prospective cohort studies and intervention trials is needed to further verify causal relationships and optimize intervention strategies, providing a more solid scientific foundation for precise and individualized CLBP prevention and treatment strategies.

## Data Availability

The original contributions presented in the study are included in the article/supplementary material, further inquiries can be directed to the corresponding authors.

## References

[1] Hoy D March L Brooks P Blyth F Woolf A Bain C . The global burden of low back pain: estimates from the global burden of disease 2010 study. Ann Rheum Dis. (2014) 73:968–74. doi: 10.1136/annrheumdis-2013-204428, 24665116

[2] Smuck M Kao MC Brar N Martinez-Ith A Choi J Tomkins-Lane CC. Does physical activity influence the relationship between low back pain and obesity? Spine J. (2014) 14:209–16. doi: 10.1016/j.spinee.2013.11.010, 24239800

[3] Geneen LJ Moore RA Clarke C Martin D Colvin LA Smith BH. Physical activity and exercise for chronic pain in adults: an overview of Cochrane reviews. Cochrane Database Syst Rev. (2017) 4:CD011279. doi: 10.1002/14651858.CD011279.pub3 28436583 PMC5461882

[4] Foster NE Anema JR Cherkin D Chou R Cohen SP Gross DP . Prevention and treatment of low back pain: evidence, challenges, and promising directions. Lancet. (2018) 391:2368–83. doi: 10.1016/S0140-6736(18)30489-6, 29573872

[5] Wong CK Mak RY Kwok TS Tsang JS Leung MY Funabashi M . Prevalence, incidence, and factors associated with non-specific chronic low Back pain in community-dwelling older adults aged 60 years and older: a systematic review and Meta-analysis. J Pain. (2022) 23:509–34. doi: 10.1016/j.jpain.2021.07.012, 34450274

[6] Heuch I Heuch I Hagen K Zwart JA. Physical activity level at work and risk of chronic low back pain: a follow-up in the Nord-Trøndelag health study. PLoS One. (2017) 12:e0175086. doi: 10.1371/journal.pone.0175086, 28394896 PMC5386240

[7] Tremblay MS Aubert S Barnes JD Saunders TJ Carson V Latimer-Cheung AE . Sedentary behavior research network (SBRN) - terminology consensus project process and outcome. Int J Behav Nutr Phys Act. (2017) 14:75. doi: 10.1186/s12966-017-0525-8, 28599680 PMC5466781

[8] Baradaran Mahdavi S Riahi R Vahdatpour B Kelishadi R. Association between sedentary behavior and low back pain; a systematic review and meta-analysis. Health Promot Perspect. (2021) 11:393–410. doi: 10.34172/hpp.2021.50, 35079583 PMC8767074

[9] Joergensen AC Strandberg-Larsen K Andersen PK Hestbaek L Andersen AN. Spinal pain in pre-adolescence and the relation with screen time and physical activity behavior. BMC Musculoskelet Disord. (2021) 22:393. doi: 10.1186/s12891-021-04263-z, 33902525 PMC8077847

[10] Guan J Liu T Gao G Yang K Liang H. Associations between lifestyle-related risk factors and back pain: a systematic review and meta-analysis of Mendelian randomization studies. BMC Musculoskelet Disord. (2024) 25:612. doi: 10.1186/s12891-024-07727-0, 39090551 PMC11293147

[11] Machado LAC Viana JU da Silva SLA Couto FGP Mendes LP Ferreira PH . Correlates of a recent history of disabling low Back pain in community-dwelling older persons: the pain in the elderly (PAINEL) study. Clin J Pain. (2018) 34:515–24. doi: 10.1097/AJP.0000000000000564, 29077624

[12] Qiu P Dong B Cao R Hu J Yang J Yu R . The relationship between physical activity levels and periodontal health status among college students: a cross-sectional study. Risk Manag Healthc Policy. (2025) 18:131–41. doi: 10.2147/RMHP.S498108, 39816784 PMC11734500

[13] Hubner FCL Telles RW Giatti L Machado LAC Griep RH Viana MC . Job stress and chronic low back pain: incidence, number of episodes, and severity in a 4-year follow-up of the ELSA-Brasil musculoskeletal cohort. Pain. (2024) 165:2554–62. doi: 10.1097/j.pain.0000000000003276., 38787636

[14] Jiang H Zhang X Liang J. The combined effect between sleep disorders and depression symptoms on chronic low Back pain: a cross-sectional study of NHANES. J Pain Res. (2024) 17:2777–87. doi: 10.2147/JPR.S471401, 39220223 PMC11363950

[15] Zhang X Niu X Wang M Li Q Feng H Wang Y . Association between physical activity trajectories and successful aging in middle-aged and elderly Chinese individuals: a longitudinal study. BMC Public Health. (2025) 25:1812. doi: 10.1186/s12889-025-23021-7, 40380161 PMC12082857

[16] Zou Z Wang Z Herold F Kramer AF Ng JL Hossain MM . Validity and reliability of the physical activity and social support scale among Chinese established adults. Complement Ther Clin Pract. (2023) 53:101793. doi: 10.1016/j.ctcp.2023.101793., 37579659

[17] Gallardo-Alfaro L Bibiloni MDM Mascaró CM Montemayor S Ruiz-Canela M Salas-Salvadó J . Leisure-time physical activity, sedentary behaviour and diet quality are associated with metabolic syndrome severity: the PREDIMED-plus study. Nutrients. (2020) 12:1013. doi: 10.3390/nu12041013 32272653 PMC7230557

[18] Fan Z Yue G Yu D Zhang M Kitaura H. The joint impact of heavy metals and polycyclic aromatic hydrocarbons on periodontitis. Int Dent J. (2025) 75:100879. doi: 10.1016/j.identj.2025.100879, 40580830 PMC12268075

[19] Cao R Qiu P Zhou Y Dong B Han Y Fan Z. The underlying relationship between exercise and the prevalence of periodontitis: a systematic review and meta-analysis. BMC Sports Sci Med Rehabil. (2023) 15:161. doi: 10.1186/s13102-023-00759-4, 38012769 PMC10683191

[20] Zhu Y Wu Y Wang Y Yang H Zhang M Zhu H . Association between exposure to blood heavy metal mixtures and overactive bladder risk among U.S. adults: a cross-sectional study. Front Public Health. (2025) 13:1597321. doi: 10.3389/fpubh.2025.1597321, 40535431 PMC12174091

[21] Quentin C Bagheri R Ugbolue UC Coudeyre E Pélissier C Descatha A . Effect of home exercise training in patients with nonspecific low-back pain: a systematic review and meta-analysis. Int J Environ Res Public Health. (2021) 18:430. doi: 10.3390/ijerph18168430, 34444189 PMC8391468

[22] Suh JH Kim H Jung GP Ko JY Ryu JS. The effect of lumbar stabilization and walking exercises on chronic low back pain: a randomized controlled trial. Medicine (Baltimore). (2019) 98:e16173. doi: 10.1097/MD.0000000000016173, 31261549 PMC6616307

[23] Grooten WJA Boström C Dedering Å Halvorsen M Kuster RP Nilsson-Wikmar L . Summarizing the effects of different exercise types in chronic low back pain - a systematic review of systematic reviews. BMC Musculoskelet Disord. (2022) 23:801. doi: 10.1186/s12891-022-05722-x, 35996124 PMC9394044

[24] Wewege MA Booth J Parmenter BJ. Aerobic vs. resistance exercise for chronic non-specific low back pain: a systematic review and meta-analysis. J Back Musculoskelet Rehabil. (2018) 31:889–99. doi: 10.3233/BMR-170920, 29889056

[25] Blanco-Giménez P Vicente-Mampel J Gargallo P Maroto-Izquierdo S Martín-Ruíz J Jaenada-Carrilero E . Effect of exercise and manual therapy or kinesiotaping on sEMG and pain perception in chronic low back pain: a randomized trial. BMC Musculoskelet Disord. (2024) 25:583. doi: 10.1186/s12891-024-07667-9, 39054514 PMC11270888

[26] Nakamura Y Nojiri K Yoshihara H Takahata T Honda-Takahashi K Kubo S . Significant differences of brain blood flow in patients with chronic low back pain and acute low back pain detected by brain SPECT. J Orthop Sci. (2014) 19:384–9. doi: 10.1007/s00776-014-0534-2, 24500293

[27] Overstreet DS Strath LJ Sorge RE Thomas PA He J Wiggins AM . Race-specific associations: inflammatory mediators and chronic low back pain. Pain. (2024) 165:1513–22. doi: 10.1097/j.pain.0000000000003154, 38323608 PMC11189762

[28] Heffner KL France CR Trost Z Ng HM Pigeon WR. Chronic low back pain, sleep disturbance, and interleukin-6. Clin J Pain. (2011) 27:35–41. doi: 10.1097/ajp.0b013e3181eef761, 21188850 PMC3058637

[29] Baraniuk JN Whalen G Cunningham J Clauw DJ. Cerebrospinal fluid levels of opioid peptides in fibromyalgia and chronic low back pain. BMC Musculoskelet Disord. (2004) 5:48. doi: 10.1186/1471-2474-5-48, 15588296 PMC539267

[30] Bontrup C Taylor WR Fliesser M Visscher R Green T Wippert PM . Low back pain and its relationship with sitting behaviour among sedentary office workers. Appl Ergon. (2019) 81:102894. doi: 10.1016/j.apergo.2019.102894, 31422243

[31] Sirucek L De Schoenmacker I Gorrell LM Lütolf R Langenfeld A Baechler M . The periaqueductal gray in chronic low back pain: dysregulated neurotransmitters and function. Pain. (2025) 166:1690–705. doi: 10.1097/j.pain.0000000000003617, 40372313 PMC12168810

[32] Klyne DM Hodges PW. Circulating Adipokines in predicting the transition from acute to persistent low Back pain. Pain Med. (2020) 21:2975–85. doi: 10.1093/pm/pnaa052, 32232467

